# Is Pilates effective in improving depressive disorders? A comprehensive overview

**DOI:** 10.1097/YIC.0000000000000541

**Published:** 2024-02-29

**Authors:** Francesca Legnani, Lorenzo Tassi, Teresa Surace, Enrico Capuzzi, Alice Caldiroli, Massimo Clerici, Massimiliano Buoli

**Affiliations:** a Department of Neurosciences and Mental Health, Fondazione IRCCS Ca’Granda Ospedale Maggiore Policlinico, Milan; b School of Medicine and Surgery, University of Milano Bicocca; c Department of Mental Health, Fondazione IRCCS San Gerardo dei Tintori, Monza (MB); d Department of Pathophysiology and Transplantation, University of Milan, Milan, Italy

**Keywords:** depressive disorders, physical exercise, Pilates

## Abstract

Depressive disorders are disabling conditions that account for high social costs. Pilates demonstrated to have several beneficial effects on health. Objective of this manuscript was to systematically review the literature about the effects of Pilates on depressive disorders. A bibliographic search was conducted in the main database sources (Pubmed, Medline, and Scopus). The inclusion criteria consisted of articles written in English language about the effectiveness of Pilates on depressive symptoms. Most of included studies are randomized controlled trials (10 out of 12). The available literature agrees in indicating that Pilates is effective in improving depressive symptoms especially when compared to inactivity and when this practice is administered for a medium-long period (8–16 weeks). In addition, Pilates seems to have at least comparable effectiveness than aerobic exercise. Pilates can be considered a reliable complementary treatment for people with depressive disorders. These findings should be interpreted considering the different types of practice administered as well as the different duration of the programs or rating scales used to assess mood symptoms. Studies with a more homogenous design are needed to confirm and make generalizable the results presented in this review.

## Introduction

Pilates is a method of exercise founded by Joseph Hubertus Pilates in the 1920s. Born in Germany, he initially developed his method in England during the internment as an enemy prisoner during the First World War, taking inspiration from his personal experience of a sickly child who dedicated much time in fitness and then became a professional boxer (Pilates and Miller, 1945). Subsequent implementation came from some contacts with the dance world when he returned to Germany and then in the USA where he finally established his business (Joyce and Kotler, 2017). He called his method ‘Contrology’, emphasizing the fact that the Pilates learning process begins in the moment of complete mental control of the exercises. The integration of mind and body is achieved with exercises that activate deep muscles: the activity can be carried out on the mat (mat Pilates) or with specific machines (reformer Pilates) (Yilmaz *et al.*, 2022). However, in the context of the Great Depression, where people’s lives were absorbed by economic issues, few people were attracted by his principle of ‘balance of body and mind’. In his lifetime, Pilates published very little about his method, primarily disseminated through apprenticeship, where learners gained knowledge directly from themselves or experienced practitioners. The first generation of teachers was trained directly by Pilates and his wife Klara Zeuner in their studio in New York, and they spread the method exactly as they had been taught (classical Pilates). The next teacher generations varied the classical method including variants of the original exercises. In 1980 an important boost to the diffusion of the method outside the USA was given by the publication of ‘The Pilates Method of Physical and Mental Conditioning’ (Friedman *et al*., 1980). The book explained the fundamentals of Pilates: (1) concentration on every single movement, (2) control of each part of the body, (3) focus on the center or ‘powerhouse’ as he called the area that forms a continuous band between the bottom of rib cage and the line across hips, (4) smoothness of movements, (5) precision and (6) deep breathing, which means full inhalation and exhalation paired with every motion. Based on these principles, Pilates stressed out the importance of spinal flattening, body lengthening, and postural control. Current styles can be divided into two basic schools: the repertory approach and modern Pilates (Lewitt *et al.*, 2019). The repertory approach closely follows the original exercises as set out by Pilates himself, and later by Friedman and Eisen. This more traditional method uses set exercise sequences and set numbers of repetitions, which can only be slightly adjusted for different body types or problems. These sequences are always performed while maintaining abdominal and postural control. A certain importance is given to the transitions between exercises and the fluidity of movements. Of note, Pilates was inspired by the movement of felines in constructing the exercise sequence. On the other hand, Modern Pilates integrates concepts from other fitness or spiritual practices such as yoga to maximize physical and mental benefit and integrate the learned concepts into daily life (Latey, 2001). Even though there are several variations of contemporary Pilates, they all belong to one of the two above-mentioned forms: “mat Pilates” and “reformer Pilates.” The former only requires a mat and bodyweight is the only resistance you work with; the latter uses a sliding platform with springs that provide adjustable resistance (Phuphanich *et al.*, 2020). Finally, it is important to specify that Pilates is not a simple physical exercise and for this reason, it deserves a separate in-depth analysis. It is based on training that involves both the mind and the body. In developing his method, Pilates himself quoted the philosopher Schiller who declared that “the mind itself shapes the body”. Learning the method only begins with complete mental control of the different exercises (Latey, 2001; Wells *et al.*, 2012).

Depression is the leading cause of mental health-related disease burden and a major cause of disability worldwide affecting approximately 280 million people and accounting for more than 47 million disability-adjusted life-years in 2019 (GBD 2019 Diseases and Injuries Collaborators, 2020). First-line treatments of this condition are represented by antidepressants (Selective Serotonin Reuptake Inhibitors and Selective Serotonin Noradrenalin Inhibitors) as well as cognitive behavioral psychotherapy in case of mild severity (Fugger *et al.*, 2022; Qaseem *et al.*, 2023). However, about a third of patients may not respond to a first-line treatment (Scala *et al.*, 2023), so antidepressants could be combined with pharmacological compounds (e.g. aripiprazole) (Caldiroli *et al.*, 2021), nutrients (e.g. ω3) (Mehdi *et al.*, 2023), psychotherapy or other non-pharmacological strategies such as neuromodulation techniques or physical exercise (Kvam *et al.*, 2016). Of note, physical exercise exerts a beneficial effect on different biological systems including immunity (Myers *et al.*, 2019; Scheffer and Latini, 2020) and metabolism that are frequently altered in patients affected by depressive disorders (Iodice *et al.*, 2021; Caldiroli *et al.*, 2022). For these reasons, physical activity has been studied in the last year as a potential treatment or a preventive measure for mood disorders (Pearce *et al.*, 2022). Even patients, who practice physical activity less than the recommended dose, show a significantly less risk of depressive disorders than those who are fully sedentary (Pearce *et al.*, 2022). Of note, several authors demonstrated the beneficial effect of Pilates on different aspects of health including musculoskeletal function (Cruz-Ferreira *et al.*, 2011) and back pain (Lim *et al.*, 2011; Pereira *et al.*, 2012) as well as fall prevention and physical fitness of elderly subjects (Bullo *et al.*, 2015). In contrast, to the best of our knowledge, limited evidence is available regarding mental health benefits of this body-mind practice. The purpose of this review was then to systematically screen literature about the effects of Pilates on depressive disorders.

## Methods

The present review was registered on the PROSPERO database (CRD42022311864). A bibliographic search was conducted by two researchers independently, in accordance with the PRISMA Guidelines for systematic reviews, using the following databases: Pubmed/MEDLINE and SCOPUS. Articles published until 13 May 2023 were included. The keywords “depressive,” “depressive symptoms”, “depression” were individually matched with the term “Pilates” [Pilates AND (depressi* OR depressive OR depressive symptom*)]. The inclusion criteria consisted of articles written in English about the effectiveness of Pilates on depressive symptoms. The exclusion criteria were the following: studies including animals, reviews and meta-analyses, articles not written in English and studies including people affected by significant medical comorbidities. We defined as significant comorbidities the medical conditions associated with prominent inflammation or cerebral lesions, thus largely affecting mood symptoms (e.g. multiple sclerosis, cancer, rheumatoid arthritis) (Cambria *et al.*, 2022). No restriction criteria were applied according to the study design: both open-label and randomized controlled studies were included. A first selection of the papers was realized according to the title, thus excluding out-of-topic papers, reviews, and meta-analyses. Following the initial screening, each paper underwent a full-text review to determine its relevance and adherence to the inclusion criteria. The screening and the extraction, as well as the full-text review, were performed by two researchers independently (FL and LT), and the articles on which there was no consensus were evaluated collectively. Articles dealing with the improvement of depressive symptoms in adults following a Pilates exercise intervention were included. According to PICOs criteria, Population: adults without severe organic comorbidities (e.g. those like multiple sclerosis that can be directly responsible for mood symptoms); Intervention: Pilates exercise; Comparison: assessment of depressive symptoms before and after a Pilates program in comparison with no exercise or other type of physical activity (e.g. walking); Outcomes: clinical improvement in depressive symptoms after the Pilates intervention (Linares-Espinós *et al.*, 2018). Finally, an evaluation of the quality of the included studies was performed according to the Qualitative Assessment Tool for Quantitative Studies (Armijo-Olivo *et al.*, 2012). This tool was chosen over other instruments such as the Cochrane risk-of-bias (designed for the quality evaluation of randomized trials) because we decided to include open-label studies in the results of the present review (Zeng *et al.*, 2015).

## Results

### General considerations

**Fig. 1:**
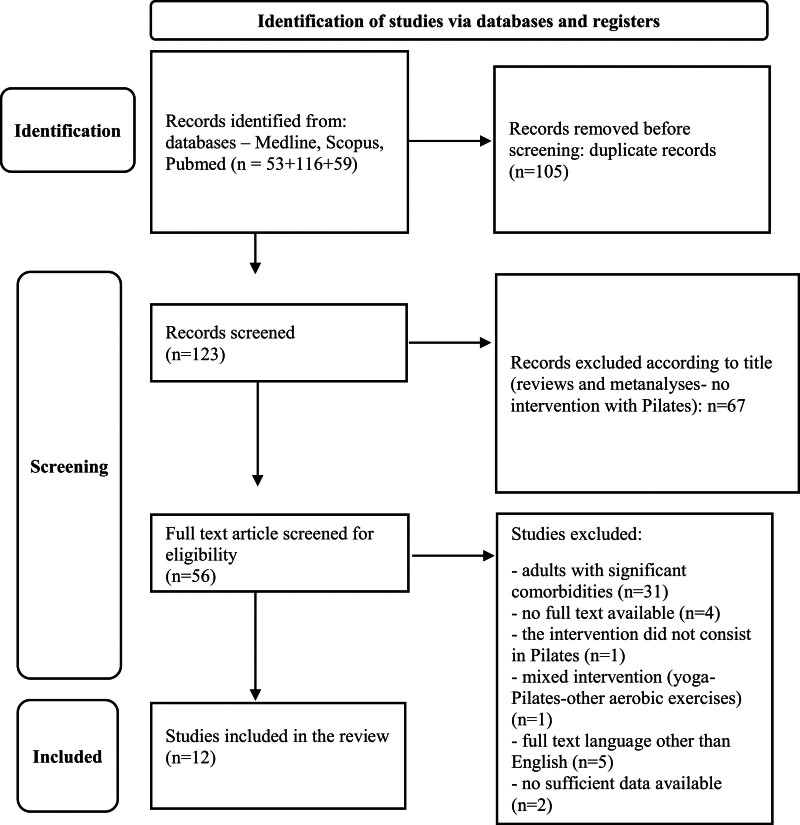
PRISMA diagram for systematic reviews.

Two hundred twenty-eight papers were initially identified, 105 were duplicated, and 111 were excluded using the above-mentioned criteria (Fig. 1). The remaining 12 articles were included and the findings are described in the present review (Table 1). In Table 2, the quality evaluation of the studies is reported. Two studies were rated as high-quality ones, the remaining half presented one aspect of weakness (moderate global rating) and half had multiple aspects of weakness. They are all studies conducted between 2016 and 2023 with a sample size varying from 16 to 148 subjects. The included articles were studies which evaluated the effect of Pilates exercise on the depressive symptoms of the participants, which were measured through psychometric scales before and after the Pilates intervention. In some studies, the participants have not been practicing any regular physical exercise for at least 6 months (Vancini *et al*., 2017; Curi *et al.*, 2018; Senturk *et al.*, 2021; Bulguroglu and Bulguroglu, 2023) or were currently inactive (Saltan and Ankarali, 2021; Soori *et al.*, 2022). In one trial the subjects had no previous Pilates experience (Fleming *et al.*, 2020), in another were not regular Pilates practitioners (sessions/week for at least 6 months in the last year) (Aibar-Almazán *et al*., 2019), while the remaining articles did not specify the presence of previous Pilates experience (Roh, 2016; Çitil and Kaya, 2021; Gong *et al.*, 2022; Kim and Hyun, 2022).

**Table 1:** Summary of the findings about the effectiveness of Pilates on depressive disorders

Study	Country	Type of study	Sample	Intervention	Outcome	Results
Roh, 2016	South Korea	16-week clinical trial with no control group	148 women > 60 years	All the enrolled women attended 3 Pilates sessions per week for a period of 16 weeks, doing mat Pilates in the first 8 weeks and band exercises in the subsequent 8 weeks	Variation in Geriatric Scale of Depression – Short form (GDS-15) after the intervention	GDS-15 scores significantly decreased after the Pilates program compared to baseline (*P* < 0.001)
Vancini *et al*., 2017	Brazil	8-week randomized controlled study	63 overweight or obese adults aged 18–66 (BMI > 25)	Participants were randomized into 3 groups: walking (n = 21), Pilates (n = 22) and control (n = 20). The walking and Pilates groups received 1 hour-sessions three times per week, while the control group did not receive any exercise program	Variation in BDI scores before and after intervention	In the Pilates group, there was a 27.5% decline of BDI scores (16.7 ± 6.8 before versus 12.1 ± 6.4 after training). Similarly, in the walking group BDI scores had a 35.2% decrease after training (18.7 ± 6.9 before versus 12.1 ± 6.7 after training) On the contrary, the reduction of BDI scores in the control group was minimal (11.9 ± 6.9 at baseline versus 9.8 ± 5.7 at endpoint)
Curi *et al.*, 2018	Brazil	16-week randomized controlled trial	61 women > 60 years	Participants were randomized into two groups: the experimental group (n = 31) performed two mat Pilates classes a week lasting 1 hour each (Classic Pilates Method for the first 2 weeks, adding other exercises for the following weeks); the control group (n = 30) did not do any exercise	Variation in the GHQ-12 total and depression subscale scores	Time × group effects in favor of the Pilates group were found for the GHQ-12 total score (*P* < 0.001) and the depression subscale (*P* = 0.002)
Aibar-Almazán *et al.*, 2019	Spain	12-week randomized controlled study	110 postmenopausal Spanish women (mean age 69.15 ± 8.94)	Participants were randomly allocated to either a control (n = 55) or a Pilates (n = 55) group. The Pilates group attended two sessions of mat Pilates of 1 hour each week for 12 weeks with increasing intensity of the exercise. The control group did not receive any training program	Variations on HADS depression subscale score	Intra- and inter-group analyses showed the efficacy of Pilates on depressive symptoms with an effect size (d) of 0.39 and 0.86, respectively
Fleming *et al.*, 2020	Ireland	Clinical trial with no control group	87 male university students (mean age 19.3 ± 3.1) with no previous Pilates experience	Students participated in a single 30-minute session of low-intensity mat Pilates for beginners	A): Variation on scores of QIDS and POMS-B, administered immediately before and 10 min after the Pilates session. B): Variation in POMS-B scores on the base of pre-intervention GAD status or depressive status (assessed through PSWQ-Penn State Worry Questionnaire- and QIDS)	A) Non-significant changes were found for depressed mood (*P* > 0.05) after Pilates session B) In post-hoc analyses Pilates resulted in significant improvements in POMS-B scores among participants with GAD (n = 28) (*P* < 0.007) and among participants with clinically significant depressive symptoms (n = 41) (*P* < 0.008)
Çitil and Kaya, 2021	Turkey	Three-month controlled clinical trial	50 female students from midwifery department of Istanbul University who had a score ≥ 88 in the Premenstrual Syndrome Scale (PMSS) were included through a non-probabilistic sampling method	Participants were divided into an experimental group (n = 25), performing 3 months of a Pilates program 3 days a week for 1 hour, and a control group (n = 25), maintaining their routine habits	Variation in PMSS scores (items for depression: depressive affect, depressive thoughts)	At baseline, there were NS differences in all the PMSS scale single-item scores in the two groups (*P* > 0.05). At the end of the exercise program, intervention group had significant lower mean scores compared to controls in all the single items of the PMSS scale (*P* < 0.05) except for fatigue item (*P* > 0.05). Comparing the PMSS mean scores between pre- and post-Pilate intervention, only in the experimental group there was a statistically significant decrease in all the items (*P* < 0.05)
Saltan and Ankarali, 2021	Turkey	12-week randomized controlled trial	105 university students aged 18–25 from Termal Vocational School, Yalova	Participants were randomically allocated into 3 groups: Pilates (n = 35), aerobic exercise (n = 35), control (n = 35). The groups were then matched for sex and age. Intervention groups were trained three times a week for 12 weeks. Control group did not perform any physical exercise	Variation in BDI score	After the intervention the BDI score reduction was significant in both the Pilates group and the therapeutic exercise group (*P* < 0.05) while not in the control group (*P* = 0.27)
Senturk *et al.*, 2021	Turkish Republic of Northern Cyprus	8-week randomized controlled trial	55 primary caregivers of special needs children, aged 18–55 and conducting a sedentary life	Participants were randomized into two groups: clinical Pilates exercise group (n = 28) doing 45–60 min Pilates sessions twice a week for 8 weeks, control group (n = 27) doing no physical exercise	Variation in BDI score	Statistically significant decrease in post-intervention BDI scores were observed only in Pilates group (*P* < 0.01) and not in control group (*P* = 0.85)
Soori *et al.*, 2022	Iran	12-week randomized controlled trial	75 Iranian women aged 60–85, inactive	Participants were randomized into 3 groups: 25 to aerobic exercise, 25 to Pilates, 25 to control group. Aerobic exercise and Pilates were practiced for 12 weeks	Variation in GHQ-28 score (items 22–28 assessing depression)	Pilates improved depressive symptoms more than aerobic exercise and inactivity (*P* < 0.05)
Gong *et al.*, 2022	China	8-week randomized controlled trial	42 adolescents suffering from Internet Addiction	Participants were randomly divided into two groups: the intervention group (n = 21) underwent narrative therapy in combination with Pilates exercise for 8 weeks, the control group (n = 21) did not receive any intervention and merely continued the routine life. Narrative group counseling was given once a week for 45–60 min each time. Pilates program consisted of 1-hour sessions performed twice a week.	Variation in the GHQ-12 anxiety and depression subscale scores	The intervention group showed a significant decrease on anxiety and depression subscale scores (*P* < 0.001), whereas no significant changes were observed in the control group (*P* = 0.886)
Kim and Hyun, 2022	South Korea	Longitudinal randomized controlled pilot study	16 pregnant women under 40 years old (24–28 weeks pregnant)	Participants were randomly allocated to Pilates (n = 8) or control (n = 8) group. The Pilates group performed an online Pilates program 50 min each session, twice a week for 8 weeks, using a real-time video chat app. The control group did not performed any exercise.	Variations in EPDS score	After 8 weeks of intervention, the control group showed significant increases in EPDS scores (*P* = 0.014), while the Pilates group showed a significant decrease in EPDS scores(*P* = 0.011)
Bulguroglu and Bulguroglu, 2023	Turkey	Randomized controlled study	58 healthy individuals aged 25–40	Individuals were randomly divided into three groups: online Pilates group (OPG n = 20), face-to-face Pilates group (FPG n = 18), and control group (CG n = 20). Pilates groups underwent 1-hour Pilates exercises in groups of three or four people for 8 weeks, 3 days a week. The control group performed breathing and relaxation exercises at home.	Changes in BDI score (Turkish version)	In both OPG and FPG there was a statistically significant decrease in BDI scores between pre and post intervention (*P* < 0.01), but not in CG

BDI, Beck Depression Inventory; CG, control group; d, Cohen’s d; EPDS, Edinburgh Postnatal Depression Scale; FPG, face-to-face Pilates group; GAD, Generalized Anxiety Disorder; GDS-15, Geriatric Scale of Depression – Short form; GHQ, General Health Questionnaire; HADS, Hospital Anxiety and Depression Scale; OPG, online Pilates group; POMS-B, Profile of Mood States – Brief Form; PSWQ, Penn State Worry Questionnaire; QIDS, Quick Inventory of Depressive Symptomatology.

**Table 2:** Evaluation of the quality of included studies

Studies	Quality rating
Roh, 2016	2
Vancini *et al.*, 2017	3
Curi *et al.*, 2018	2
Aibar-Almazán *et al.*, 2019	1
Fleming *et al.*, 2020	3
Çitil and Kaya, 2021	3
Saltan and Ankarali, 2021	1
Senturk *et al.*, 2021	2
Gong *et al.*, 2022	3
Kim and Hyun, 2022	3
Soori *et al.*, 2022	2
Bulguroglu and Bulguroglu, 2023	2

Global rating was performed according to these criteria (Qualitative Assessment Tool for Quantitative Studies):

1) Selection bias (sample size power and number of subjects who agreed to participate into the study).

2) Study design (randomized versus non-randomized trials).

3) Confounders (yes/no).

4) Blinding (yes/no).

5) Data collection methods (self-reported data, observations by investigators or medical records).

6) Presence of description of numbers and reasons for withdrawals and drop-outs.

1 = strong (no weak ratings according to above criteria).

2 = moderate (1 weak rating according to above criteria).

3 = weak (2 or more weak ratings according to above criteria).

### Psychometric tools

A large variety of tools were used in the studies included in this review to identify depressive symptoms in the samples. Of note, four trials (Vancini *et al.*, 2017; Saltan and Ankarali, 2021; Senturk *et al.*, 2021; Bulguroglu and Bulguroglu, 2023) assessed the severity of depressive symptoms using the Beck Depression Inventory Score (BDI), a 21-item psychometric scale frequently administered in non-psychiatry contexts (Wang and Gorenstein, 2013). In one trial (Soori *et al.*, 2022) the researchers used the General Health Questionnaire – 28, a psychometric scale organized into 28 items, assessing four different health aspects (1–7 physical symptoms, 8–14 anxiety, and insomnia, 15–21 social function, 22–28 depression) (Sterling, 2011). In one study (Fleming *et al.*, 2020) the Quick Inventory of Depressive Symptomatology (Rush *et al*., 2003) and the 30-item Profile of Mood States – Brief Form (Kuboki *et al.*, 1993) were employed by the authors to assess the severity of feelings of tension, depressed mood, and fatigue. In one trial (Aibar-Almazán *et al.*, 2019) the presence of depressive symptoms was assessed using the Hospital Anxiety and Depression scale (Smarr and Keefer, 2011). The researchers of one article (Roh, 2016) employed the Geriatric Scale of Depression (Benedetti *et al*., 2018) to measure the severity of mood symptoms of the participants. The authors of one manuscript (Kim and Hyun, 2022) evaluated the depressive status of the sample using the Edinburgh Postnatal Depression Scale, a simple tool to assess mental health during the perinatal period (Serati *et al.*, 2021). The General Health Questionnaire 12 (Oliveira *et al.*, 2023), which includes one item about depression, was employed to assess mood disorders and general well-being in two articles (Curi *et al.*, 2018; Gong *et al.*, 2022). Finally, Çitil and Kaya (2021) identified mood symptoms in their sample focusing on depression items of the Premenstrual Syndrome Scale (Çitil and Çitil Canbay, 2023).

### Uncontrolled studies

Two of the included articles evaluated the effect of Pilates exercise intervention on depressive symptoms without a control group. The results of one study showed a significant amelioration of depressive symptoms in a sample of elderly women after a 16-week Pilates program (Roh, 2016). Another trial with multiple method weaknesses focused on the effects of Pilates practice on depressive symptoms in young adult males, who received a single 30-minute Pilates session. Globally, the study failed to find a significant improvement in depressive symptoms after a single Pilates session, but the magnitude of improvement increased proportionally to the baseline severity of mood symptoms (Fleming *et al.*, 2020).

### Controlled studies without an active comparator

Six mixed-quality articles have a randomized controlled design and compare the variation of depressive symptoms between participants (people who received a Pilates training program) and controls. In all these studies the total sample showed moderate depressive symptoms before starting the program and some potential participants were excluded for medical (Curi *et al.*, 2018; Aibar-Almazán *et al.*, 2019; Çitil and Kaya, 2021; Senturk *et al*., 2021; Gong *et al.*, 2022; Kim and Hyun, 2022) or social reasons (involvement as caregiver) (Senturk *et al.*, 2021). The study by Kim and coauthors specified that randomization was performed by computer (Kim and Hyun, 2022). Globally, considering all the above-mentioned studies, the rate of adherence to the program was high once it started: ≥80% in both the Pilates and control groups.

Senturk and collaborators found a significant improvement (*P* > 0.01) of depressive symptoms in healthy adults after 40–60-minute Pilates sessions, twice a week for 8 weeks (Senturk *et al.*, 2021). Aibar-Almazán *et al.* realized a high-quality study, demonstrating that a 12-week Pilates program (two 1-hour Pilates sessions per week) had a significant impact on anxious and depressive symptoms of postmenopausal women (Aibar-Almazán *et al.*, 2019). Similar positive results were reported by a study assessing the effect of a 16-week Pilates program (two mat Pilates classes lasting 1 hour each per week) on women older than 60 years (Curi *et al.*, 2018). Çitil and Kaya analyzed the impact of Pilates on premenstrual syndrome (PMS) in a sample of young women, demonstrating a significant improvement of the depressive symptoms in the Pilates group compared to controls (Çitil and Kaya, 2021). Moreover, Pilates was found to be effective in ameliorating affective symptoms and insomnia in pregnant women (Kim and Hyun, 2022). Finally, Gong and collaborators confirmed that this type of physical exercise is beneficial in adolescents presenting anxiety and depressive symptoms (Gong *et al.*, 2022).

### Controlled studies with an active comparator

Three mixed-quality articles were randomized controlled trials comparing the variation of depressive symptoms between people who practiced Pilates, controls, and an active comparator group (subjects who received an exercise/therapeutic intervention different from Pilates). In all these studies the total sample showed moderate depressive symptoms before starting the program and some potential participants were excluded for medical reasons (Vancini *et al.*, 2017; Saltan and Ankarali, 2021; Soori *et al.*, 2022). Only Vancini and coauthors (Vancini *et al*., 2017) specified that offered walking or Pilates programs to the subjects randomized to control group at the end of the study.

Soori *et al.* found that Pilates practitioners reported more improvement of depressive symptoms than inactive subjects or those practicing aerobic exercise (Soori *et al.*, 2022). Another trial focused on the effects of Pilates on affective symptoms and quality of life of individuals affected by overweight or obesity. The Pilates group received 8 weeks of exercise, three times per week, 60 min/session. Pilates demonstrated to be as effective as walking in ameliorating depressive symptoms and superior to inactivity (Vancini *et al.*, 2017). Saltan and Ankarali conducted a similar high-quality study on university students and they evaluated the effect of Pilates (three times a week for 12 weeks) on depressive symptoms (together with pain, functionality status, and quality of life), compared to inactivity or another form of therapeutic exercise, showing a significant improvement of depressive symptoms in the two active groups, without a significant difference between Pilates and therapeutic exercise (Saltan and Ankarali, 2021).

### Controlled studies assessing different Pilates methods

One study (Bulguroglu and Bulguroglu, 2023) compared the efficacy of two different ways of practicing Pilates in reducing depressive symptoms (online programs or face-to-face Pilates versus inactive controls), showing that both Pilates interventions were associated with a significant amelioration of mood symptoms differently from inactive controls. The total sample had a mean baseline BDI score corresponding to mild depression.

## Discussion

All studies agree in underlining the effectiveness of Pilates in improving depressive symptoms, apart from one study which considered the effects of a single short session (Fleming *et al.*, 2020). In the other studies, the practice is expected to be carried out 2–3 times a week for a period of between 8 and 16 weeks. This shows that Pilates shows greater effectiveness on mental conditions in case of frequent and prolonged exercise over a certain period (Grasdalsmoen *et al.*, 2020). Some authors reported how Pilates can have a beneficial effect on various biological systems including an improvement of excessive inflammation (Balogh *et al.*, 2022) or stabilization of blood pressure (Batista *et al.*, 2022). In this sense, this practice could act on some biological alterations typical of the depressive state such as immune alterations and excessive oxidative state (Caldiroli *et al.*, 2022).

Other authors have previously attempted to summarize the available literature on the effects of Pilates on depressive disorders. The meta-analytic review by Meikis and coauthors (Meikis *et al.*, 2021) focused on psychological well-being (including mood state) of healthy elderly and those with medical clinical conditions. The authors concluded that Pilates is able to improve psychological health of elderly regardless of their health condition (Meikis *et al.*, 2021). In a recent meta-analysis (Ju *et al.*, 2023) the authors decided for different inclusion criteria, in some cases including articles focusing on functional level or postural alignment of women with chronic conditions. Again, Pilates demonstrated to improve depression in women with chronic medical conditions such as cancer (Ju *et al.*, 2023). Therefore, the available data would suggest, in line with our observations, that Pilates is a versatile method, suitable for different ages and health conditions, which is capable of improving the depressive state of a wide range of subjects.

### Evidence limitations

A series of aspects of the available literature need to be discussed. The samples of the studies analyzed in this review are extremely heterogeneous in terms of several clinical characteristics potentially affecting the presence and severity of depressive symptoms, including sex (Ceresa *et al.*, 2022), age (Buoli *et al*., 2023), presence of medical comorbidity (Arnaud *et al.*, 2022) and perinatal/perimenopausal period (Ozdemir *et al.*, 2020). The effectiveness of Pilates on depressive symptoms was evaluated on healthy young adults (Fleming *et al.*, 2020; Saltan and Ankarali, 2021; Bulguroglu and Bulguroglu, 2023) or subjects vulnerable to depressive conditions such as fragile subjects’ caregivers (Senturk *et al.*, 2021), adolescents affected by Internet Addiction (Gong *et al.*, 2022), post-menopausal (Aibar-Almazán *et al.*, 2019) or pregnant women (Kim and Hyun, 2022), women with premenstrual syndrome (Çitil and Kaya, 2021), elderly subjects (Roh, 2016; Curi *et al.*, 2018; Soori *et al.*, 2022), individuals affected by obesity (Vancini *et al.*, 2017). Differences in the target population also influence how Pilates sequences are administered (Byrnes *et al.*, 2018). If, in some cases, sessions were proposed with the use of facilitators or with precise sequences of exercises (Roh, 2016; Vancini *et al.*, 2017), in others the standard mat-Pilates sequence was administered (Curi *et al.*, 2018; Aibar-Almazán *et al.*, 2019; Kim and Hyun, 2022). In addition, in some cases Pilates was guided by certificated trainers (Vancini *et al.*, 2017; Curi *et al.*, 2018; Fleming *et al.*, 2020; Aibar-Almazán *et al.*, 2019; Gong *et al.*, 2022; Kim and Hyun, 2022), while in other studies by health professionals such as physiotherapists (Senturk *et al.*, 2021; Bulguroglu and Bulguroglu, 2023). Other aspects that should be considered in the interpretation of the results of the included study are (1) the fact that sample sizes of some studies are small; (2) different tools were used to assess the presence and severity of depressive disorders (in some cases specific for special populations such as pregnant women or elderly individuals); (3) the trials were conducted in different countries where the cultural context or the organization of mental health services may have influenced the course of depressive disorders; (4) some of the included researches were conducted during COVID pandemic that had a large impact on mental health of general population and health professionals (Tabano *et al.*, 2023).

### Review limitations

In addition to the potential confounders of the included studies, some limitations of the present review should be listed. First of all, we referred to depressive disorders and not to a specific diagnostic category such as Major Depressive Disorder. Second, we decided to include uncontrolled studies which have the defect of reporting less reliable data but have the advantage of being more in line with clinical practice. Third, it was impossible to perform a meta-analytic analysis due to the extreme heterogeneity of the included studies (first of all in the tools used to measure the severity of depressive symptoms).

## Conclusion

In conclusion, Pilates appears to be a technique that seems to improve depressive symptoms and can be offered to a large audience of individuals. Research in this field is still preliminary, but it would be useful to think of a sequence of target exercises for depressive symptoms with the possible use of facilitative tools (e.g. bands) for special populations as also recommended by Pilates trainers (Lim and Hyun, 2021). The extreme variability of the clinical characteristics of the samples of the available literature supports the fact that Pilates is a flexible technique that can be beneficial on mental health of a large part of population without excessive costs (Pereira *et al*., 2022). In this regard, it will also be interesting to evaluate how the application of these exercises can improve the response to depressive treatment as demonstrated for aerobic exercise (Belvederi Murri *et al*., 2015). In contrast, like all interventions, Pilates could have undesirable effects such as excessive physical exercise which could characterize young subjects with eating disorders (Calogero and Pedrotty, 2004) in frequent comorbidity with depressive disorders (Lian *et al*., 2017). Future studies will need to clarify whether (1) this technique can be suitable for patients with different depressive severity; (2) offers benefits over other types of exercise (Franco *et al*., 2023); (3) can be specifically adapted with a series of target exercises for subjects with depressive disorders.

## Acknowledgements

Massimiliano Buoli is a certified level I Pilates instructor.

### Conflicts of interest

There are no conflicts of interest.
